# LMD-YOLO: A lightweight algorithm for multi-defect detection of power distribution network insulators based on an improved YOLOv8

**DOI:** 10.1371/journal.pone.0314225

**Published:** 2025-02-21

**Authors:** Weiyu Han, Zixuan Cai, Xin Li, Anan Ding, Yuelin Zou, Tianjun Wang

**Affiliations:** 1 School of Computer Science and Technology, Xinjiang University, Urumqi, Xinjiang, China; 2 Changzhou Open University, Changzhou, Jiangsu, China; 3 State Grid Xinjiang Electric Power Co., LTD., Urumqi, Xinjiang, China; SRM Institute of Science and Technology (Deemed to be University), INDIA

## Abstract

Insulator defect detection is a critical task in distribution network inspections. To address issues such as low detection accuracy, high model complexity, and large parameter counts caused by the variety of insulator defect types, this study propose a lightweight multi-defect detection network, LMD-YOLO, based on YOLOv8. The network improves the backbone by introducing SCConv module to improve C2f module, which reduces spatial and channel redundancy, lowering both computational complexity and the number of parameters. The SimAM attention mechanism is integrated to suppress irrelevant features and enhance feature extraction capabilities without adding extra parameters. The SIoU loss function is used in place of CIoU to accelerate model convergence and improve detection accuracy. Additionally, this study creates a target detection dataset that encompasses four types of insulators: insulator, absent insulator, broken insulator, and shedding insulator. Experimental results show that LMD-YOLO achieves a 2% higher average accuracy on the insulator dataset compared to YOLOv8n, with a 24.6% reduction in model parameters, offering an effective solution for smart grid inspections.

## Introduction

Insulators, as crucial components in the distribution grid, are exposed to complex outdoor environments for extended periods and are susceptible to defects and failures due to various factors [1]. If damaged components are not promptly identified and addressed, it can directly impact the stable operation of the power system. Statistics indicate that insulator failures contribute significantly to power equipment failures, posing a direct threat to the safe operation of the power system.

With the deep integration of artificial intelligence and transmission business, the use of drone technology for image recognition has become an indispensable tool for addressing issues with traditional manual patrol methods such as long duration, limited coverage range, and environmental constraints. UAV inspection operations require high real-time operational demands [2]. Achieving lightweight deployment of complex network models on embedded edge computing devices with existing hardware configurations while completing low computational algorithm deployment and efficient identification of various defects is essential [3]. Therefore, model algorithms need to be balanced in terms of detection accuracy and parameter quantity.

The following are the main contributions of this work:

- Enhance the C2f_ScConv feature extraction module by integrating a space and channel reconstruction convolutional module into the original C2f network structure, resulting in lightweight improvements while maintaining excellent performance. This upgrade incorporates channel and spatial information to enhance the network’s capability in extracting effective image features of insulators.
- The SimAM module is integrated within the neck and backbone networks of the LMD-YOLO model without introducing additional learning parameters. This facilitates the reduction of interference from complex environments and the improvement of the representation of defect images through reinforcing category feature extraction.
- During network training, replace the CIoU loss function with SIoU loss function, introducing the angle between real bounding box and predicted bounding box as a convergence influencing factor to expedite model convergence.
- Introduce Dysample, a highly lightweight and efficient dynamic up-sampler designed to improve the ability to identify small insulator defects in low-quality images.

## Related work

The current approach to insulation defect detection using computer vision is mainly categorized into traditional image recognition methods and deep learning techniques. The use of traditional image recognition methods, such as that employed by Zhai et al. [4], leveraged the distinctive color and spatial features of glass and ceramic insulators to pinpoint the area of insulator defects. Chen et al. [5] introduced an edge-based insulator string image segmentation method for identifying insulator faults. Yan Kang et al. [6]. integrated histogram equalization enhancement with the two-dimensional Ostu thresholding method to extract features from images and train neural networks to identify the hydrophobicity level of insulators [7]. Most conventional insulator detection methods incorporate edge detection, color features, etc. which are significantly influenced by environmental background and lack robustness, making it challenging to deploy them onto embedded devices that can meet real inspection requirements in smart grid patrols.

The recent years have witnessed the widespread adoption of deep learning-based object detection algorithms, such as YOLO (You Only Look Once) [8] series, driven by convolutional neural networks for detecting insulation defects. There are two primary approaches in object detection research: Two-stage detection networks, such as Fast R-CNN [9] and Faster R-CNN [10], are renowned for their high accuracy albeit at the cost of speed; Conversely, single-stage networks like YOLO and SSD (Single Shot Detector) [11], are known for their swiftness but with a trade-off in accuracy. The YOLO series currently stands as one of the most mature and widely used object detection algorithms. Liu Xingmou et al. [12] improved the balance cross-entropy of the YOLOv4 network and incorporated a spatial pyramid pooling (SPP) structure before and after convolution layers to enhance network detection accuracy. Xue Qiang et al. [13] enhanced transmission line defect detection performance by integrating attention mechanism into YOLOv5s backbone network while using BiFPN instead of original feature pyramid structure. Wang et al. [14] proposed an efficient and rapid feature processing module based on the FasterNet module, achieving full fusion of shallow and deep features. Moreover, they integrated BiFormer, a dynamic sparse attention into network can effectively improve the detection performance of small objects without the need to increase the network size or parameters. Hao et al. [15] propose an improved network ID-YOLO, which is based on YOLOv4 and incorporates the cross-stage partial and residual split attention network (CSP-ResNeSt). This modification aims to tackle the challenge of complex background interference in aerial images, the average accuracy of network detection is 3.5% higher than that of YOLOv4, attaining 95.63%. Hu et al. [16] implemented an innovative adaptive weight distribution multi-head self-attention module in YOLO and incorporated a CBAM attention mechanism into the backbone network to improve the detection accuracy of smaller target defects. Although such methods enrich the insulator defect detection objects, the detection scenarios are relatively simple, and the insulators are all detected in the same scenario, resulting in poor generalization of insulator defect detection in different scenarios. Based on yolov8, Chen et al. [17] integrated the GSConv module into both the backbone network and neck network, while also implementing the lightweight Content-Aware Feature Recombination (CARAFE) structure to minimize network model parameters. This approach aims to improve fusion between shallow and deep feature maps, ultimately enhancing detection accuracy within the network.

Although the above studies have attained remarkable outcomes in the detection of insulators and defects, and optimizing the model structure has improved the detection accuracy, the majority of these studies mainly concentrate on the absent of insulators, with relatively limited attention given to other types of defects. Nevertheless, these models are frequently capable of identifying only a single type of insulation defect and are unable to effectively strike a balance between improving the network detection accuracy and the model complexity in a complex and diverse context. In the actual UAV inspection operation, it is necessary to fulfill not only the requirements of the model detection performance but also the requirements of the hardware platform resource allocation and deployment [18]. Compared with the single and repetitive background in the insulator public dataset, the background of the distribution network is complex and encompasses various types of insulator defects, resulting in a relatively low effective recognition rate of the model. To address these issues, this paper proposes a lightweight multi-defect detection network LMD-YOLO based on the YOLOv8 structure. By minimizing the quantity of model parameters and evaluating model detection performance, the method integrates diverse insulator defect types while achieving a balance between detection accuracy and model size.

## Materials and methods

### Dataset

The insulator defect images used in this paper consist of the open data set CPLID (Chinese Power Line Insulator Dataset) [19], the open insulator defect dataset released by the Intelligent Transmission and Distribution Institute of Shanghai Jiao Tong University [20], and real insulator defect pictures from Xinjiang power grid inspected by UAV, totaling 1951 images. The training and validation sets are annotated using the Labeimg tool to generate a label file that adheres to the YOLO data set format.

The Table 1 above illustrates the target data distribution for insulators. The training set, test set, and validation set were divided in a ratio of 7:1.5:1.5.

**Table 1:** Distribution of insulator target data.

Target	Insulator	Absent Insulator	Broken Insulator	Shedding Insulator
Totals	3560	828	413	2243

**Fig 1:**
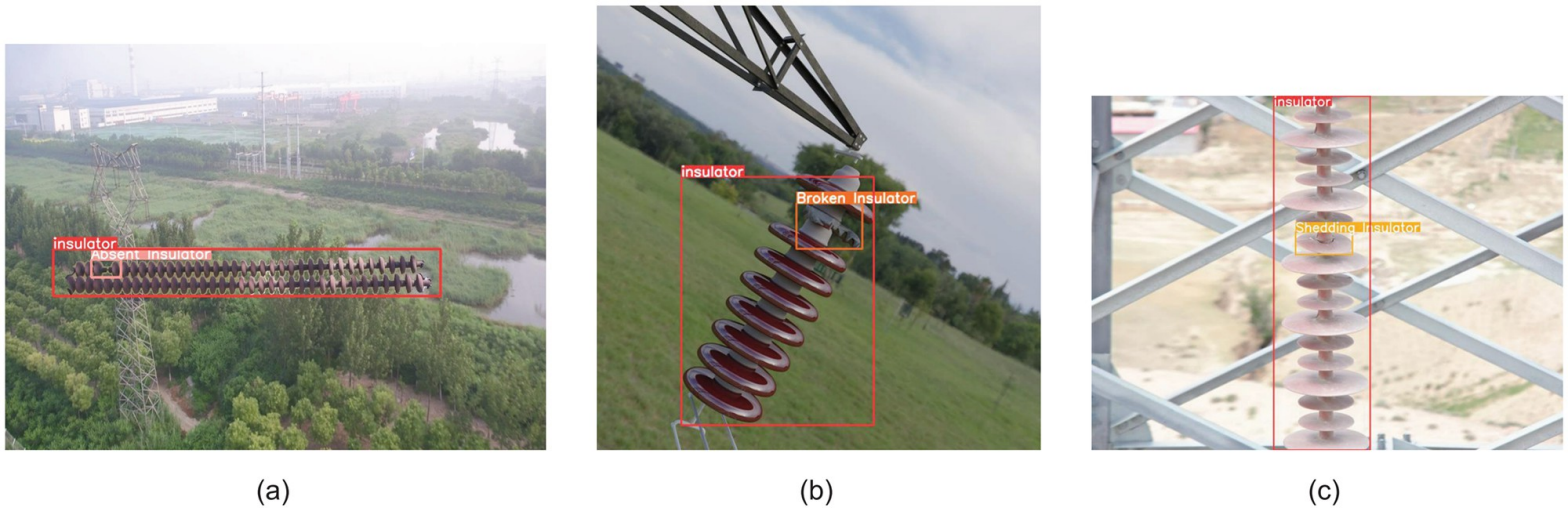
**(a)** shows ‘Insulator’ and ‘Absent Insulator’, **(b)** presents ‘Insulator’ and ‘Broken Insulator’, and **(c)** depicts ‘Insulator’ and ‘Shedding Insulator’.

The insulators were labeled according to four categories: ‘Insulator’, ‘Absent Insulator’, ‘Broken Insulator’, and ‘Shedding Insulator’. Part of the sample content is shown in Fig 1. The following describes the different types of insulator defects:

- Shedding insulator refers to the situation where the insulator has not completely detached from its fixed position but has loosened or partially deviated from the fixing device. Common causes include mechanical stress, improper installation, and material aging.
- Broken insulator means that there are cracks, fractures or fragmentation in the ceramic, glass or composite parts of the insulator, which may be caused by mechanical impact, temperature variation or manufacturing defects. Broken insulators can compromise their ability to insulate, raise the level of leakage current, and potentially result in breakdown incidents, significantly impacting the secure and consistent functioning of the power system.
- Absent insulator refers to the situation where the insulator in the power line has completely separated from its original position and no longer exists at the fixed point due to various reasons. This may be caused by improper installation, extreme weather, external damage or long-term lack of maintenance. The absence of insulators will directly result in the loss of necessary insulation protection for the power line, increase the risk of electrical accidents, and seriously affect the safety and stability of power transmission.

**Fig 2:**
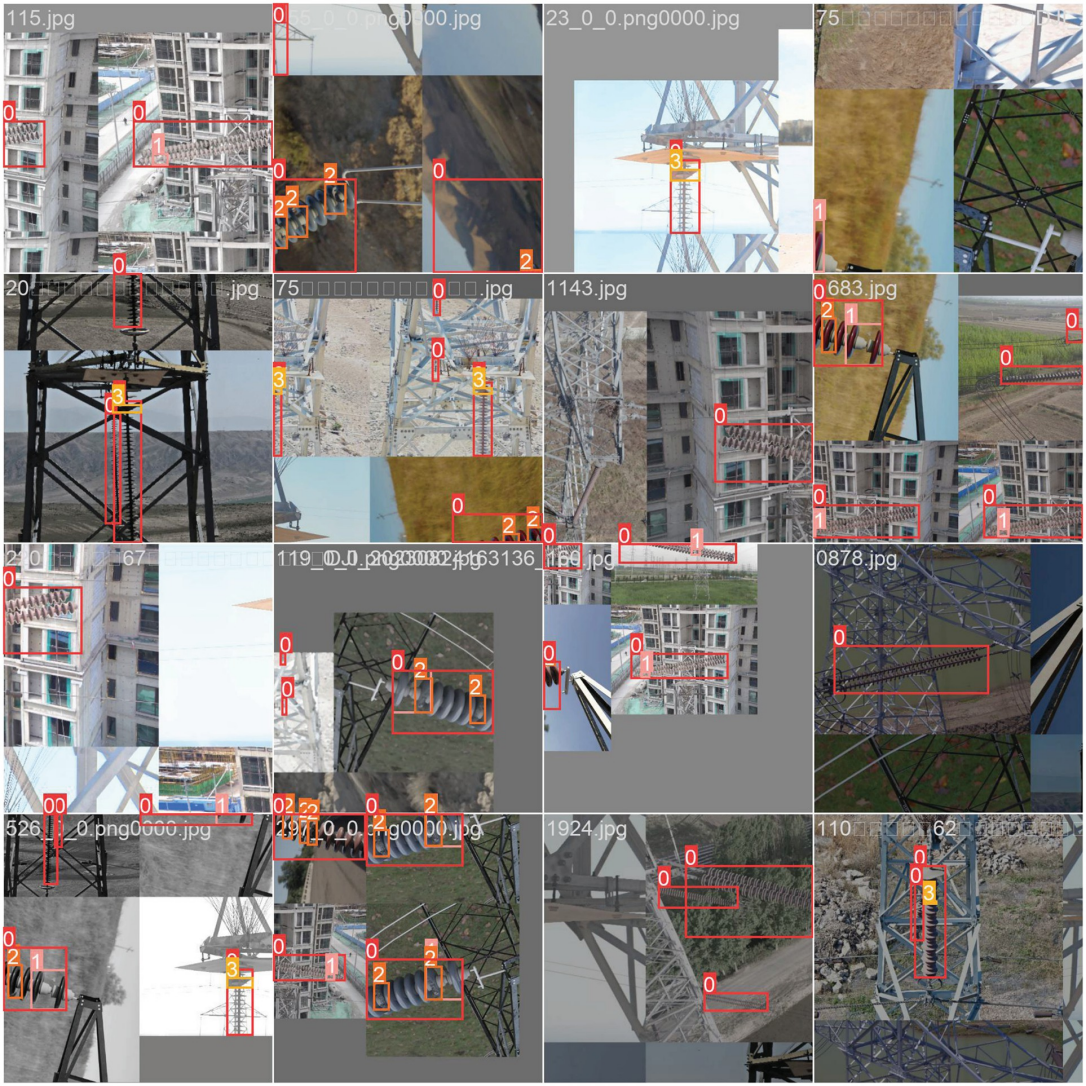
The image shows the detection results after data augmentation, with red boxes marking targets in various scenes. Data augmentation methods include mosaic, flipping and other transformations.

In order to improve the model’s capacity for generalization and reduce the likelihood of overfitting, a dynamic data augmentation approach was utilized throughout the training phase, including flipping, brightness changes, contrast changes, Mosaic, and erasing. Fig 2 displays some images following data augmentation.

### Baseline model YOLOv8

**Fig 3:**
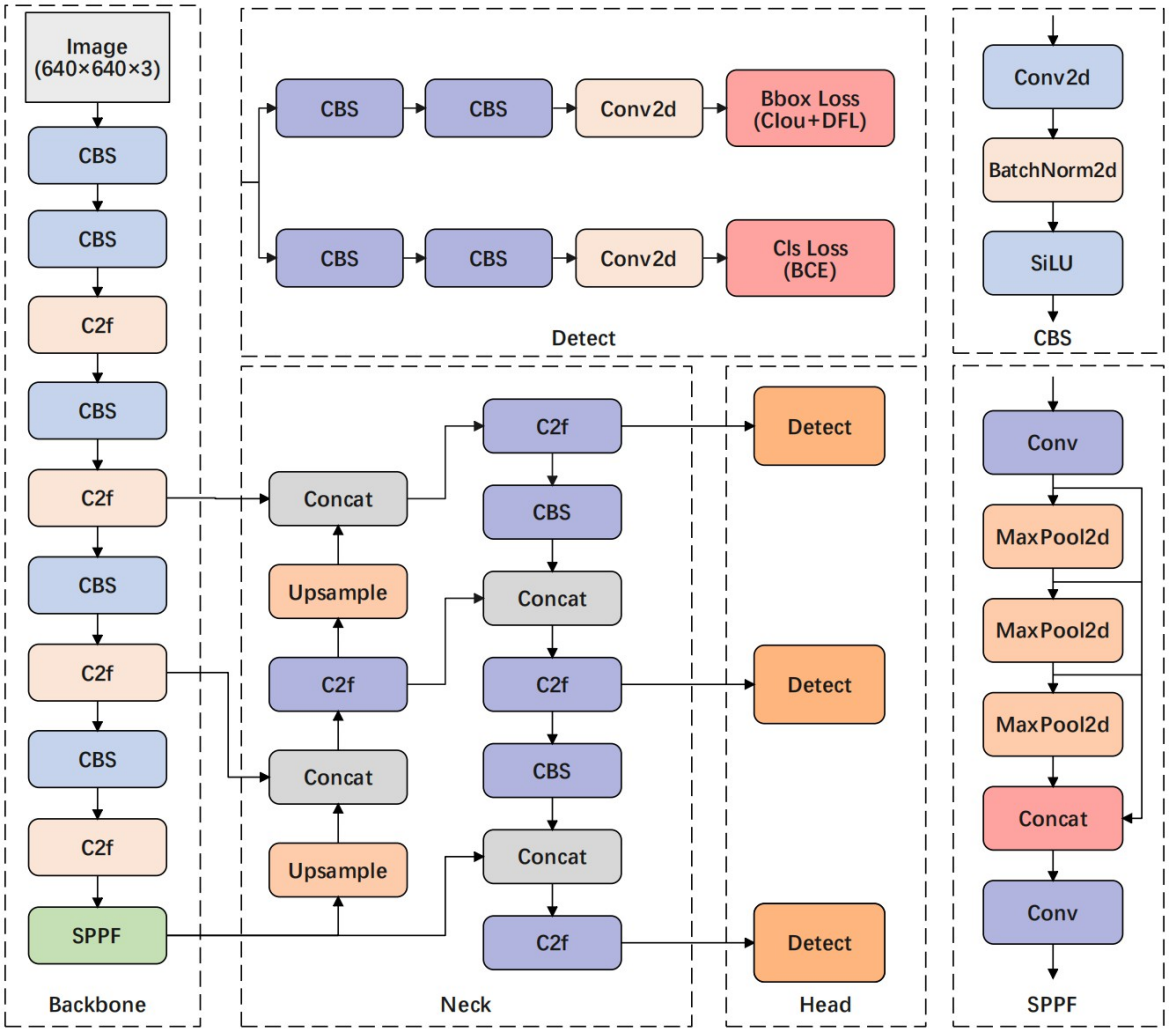
Network structure diagram of YOLOv8 algorithm.

The YOLOv8 network, unveiled as the most advanced model in 2023, enhances overall detection performance by optimizing the network structure while leveraging the strengths of its predecessors. Its widespread adoption for various object detection tasks is attributed to its efficient real-time processing and network architecture. As shown in Fig 3, The network consists of three primary elements: the backbone network, neck network, and detection head. The backbone network functions as a module for extracting features, responsible for extracting initial features from input images at different scales. In comparison to YOLOv5, both the backbone and neck networks have been enhanced by adopting C2f structure [21] instead of YOLOv5’s C3 structure and adjusting channel numbers for models at different scales to achieve lightweighting and richer gradient flow information. The neck network, positioned between the backbone network and the detection head, combines feature information extracted from the backbone to produce more detailed data, leading to the creation of three additional feature maps. The detection head utilizes these features for prediction while replacing previous versions’ Anchor-Base approach with an Anchor-Free method to generate final outputs. Due to its introduction of new functionalities and improvements that enhance performance and flexibility while providing models at different scales to meet diverse scene requirements, YOLOv8 has gained extensive application within computer vision research.

### LMD-YOLO algorithm

**Fig 4:**
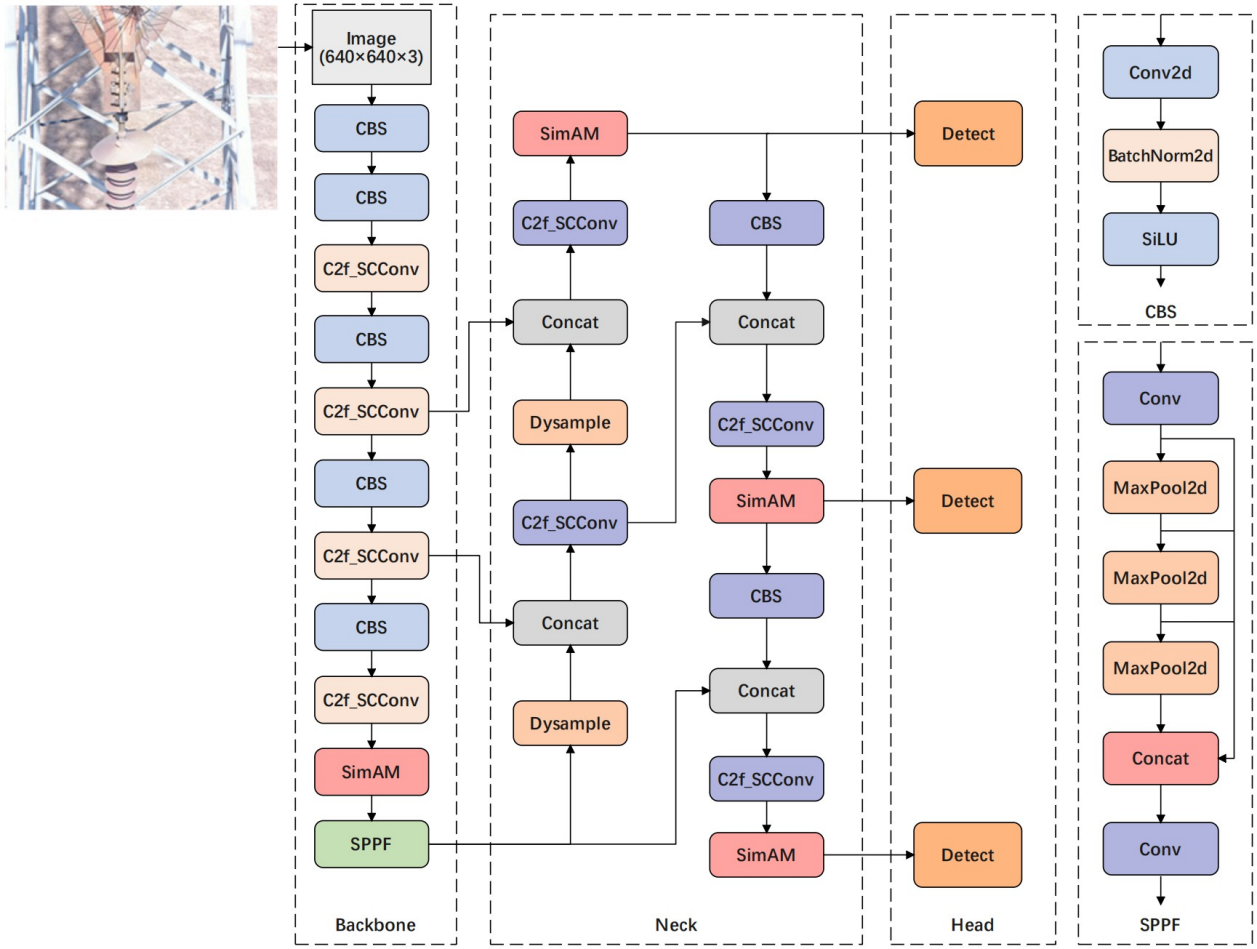
Network structure diagram of LMD-YOLO algorithm. The C2f module in both the backbone network and the neck network has been substituted with C2f_SCConv. Additionally, SimAM attention has been incorporated to improve the capability of focusing on insulator defects. Lastly, dysample has replaced upsample in the original network.

The enhanced network structure is illustrated in the Fig 4. Initially, we integrated the spatial and channel reconstruction convolutional module with the C2f module to improve the capacity for extracting intricate features while reducing parameter count. Subsequently, an effective SimAM attention module was introduced to evaluate feature weights and diminish interference from complex insulator backgrounds. In a later stage, Dysample has been implemented to replace the simple upsampling in the original feature fusion neck structure, resulting in improved model efficiency and performance for insulator defect detection, all while reducing computational costs. Finally, during network training, we substituted the CIoU loss function in Yolov8 baseline model with SIoU loss function by considering the orientation angle between predicted bounding box and target bounding box to expedite model convergence and enhance its performance.

#### C2f_SCConv

**Fig 5:**
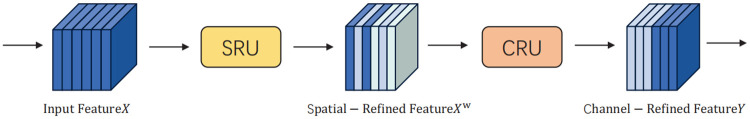
Network structure of SCConv. The SCConv design integrates the spatial reconstruction unit (SRU) and channel reconstruction unit (CRU).

To enable the deployment of lightweight networks on embedded devices, this study introduces SCConv convolution to optimize the LMD-YOLO model. The Spatial and Channel reconstruction Convolution (SCConv) [22], proposed in 2023, serves as a novel convolutional module that replaces standard convolution to minimize redundant computations and facilitate representative feature learning, thereby reducing model parameters and floating-point operations (FLOPS). As shown in Fig 5, the network structure of SCConv.

**Fig 6:**
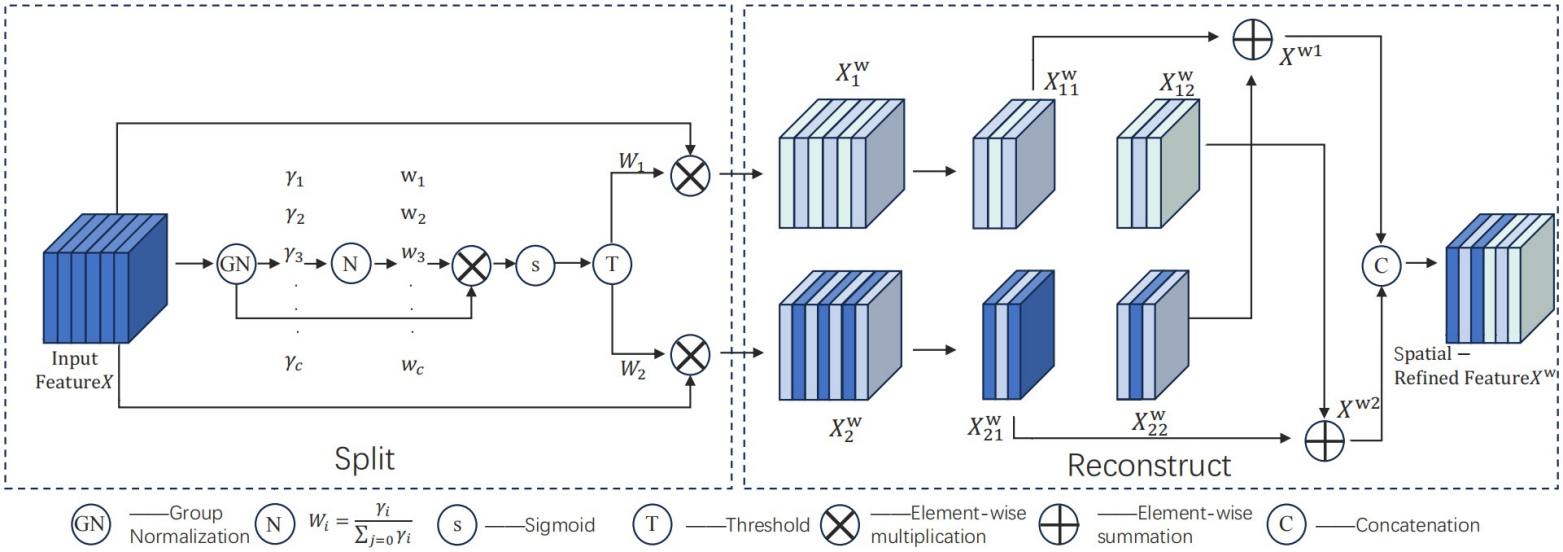
Structure of SRU.

The SRU effectively mitigates spatial redundancy through separation-reconstruction operations, depicted in Fig 6, enhancing computational efficiency within the model. By evaluating information content across different feature maps using group normalization [23] scaling factors, the separation operation segregates spatially rich feature maps from sparse ones, yielding feature map *W*_1_ with higher information density and redundant feature map *W*_2_ with lower information density. Subsequently, these enriched features *W*_1_ are integrated with sparse features *W*_2_ to generate significant information $X_{1}^{w}$ and $X_{2}^{w}$; cross-reconstruction is then employed to combine these distinctively rich features into the final *X*^*W*^ feature map following spatial reconstruction. The whole process of Reconstruct operation can be expressed as:

$$\begin{array}{c} \left\{ \begin{array}{cc} & X_{1}^{w}=W_{1}⊗X\\ & X_{2}^{w}=W_{2}⊗X\\ & X_{11}^{w}⊕X_{22}^{w}=X^{w1}\\ & X_{21}^{w}⊕X_{12}^{w}=X^{w2}\\ & X^{w1}\cup X^{w2}=X^{w} \end{array}\right. \end{array}$$ (1)

The symbol ⊕ denotes element-wise addition, ∪ represents concatenation operation.

**Fig 7:**
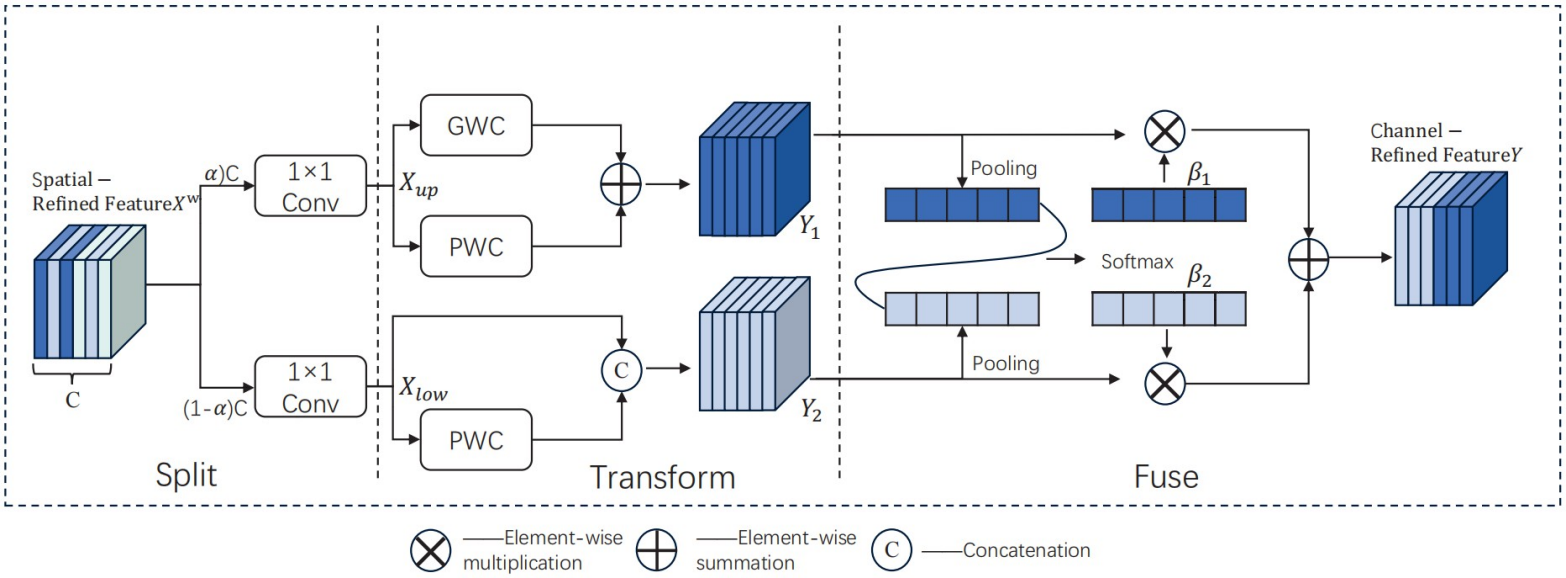
Structure of CRU.

The CRU (Channel Reconstruction Unit) includes three operations: segmentation, transformation, and fusion, as shown in the Fig 7. For a given spatial fine feature $X^{w}\in R^{\left(c\times h\times w\right)}$, the *X*^*w*^ channel is divided into *αC* channels and (1 − *α*)*C* channels, where 0 ≤ *α* ≤ 1. Subsequently, a 1 × 1 standard convolution is employed to compress the feature map’s channels, further enhancing computational efficiency. Following this, by controlling the squeeze ratio *r* to adjust the size of CUR feature channels after performing segmentation and compression operations, the spatially refined feature *X*^*w*^ is partitioned into upper part *X*_*up*_ and lower part *X*_*low*_. Notably, *X*_*up*_ functions as a rich feature extractor inputted into the upper transformation stage; group-wise convolution (GWC) [24] and point-wise convolution (PWC) are utilized to replace standard *K* × *K* convolutions for extracting features with reduced computational cost. Meanwhile, PWC is applied to *X*_*low*_ in order to generate a feature map with shallow hidden details. The generated feature map is then concatenated with the feature map *X*_*low*_ Obtain the feature Fig *Y*_2_. The specific calculation process is shown in Eqs 2 and 3.

$$\begin{array}{c} Y_{1}=\text{CWC}(X_{up})+\text{PWC}\left(X_{up}\right) \end{array}$$ (2)

$$\begin{array}{c} Y_{2}=X_{\text{low}}\cup \text{PWC}\left(X_{\text{low}}\right) \end{array}$$ (3)

After the conversion operation, an adaptive method is utilized to integrate the output features *Y*_1_ and *Y*_2_ from the upper and lower conversion stages. Subsequently, global average pooling is employed to process *Y*_1_ and *Y*_2_ in order to gather global spatial information along with channel statistics $S_{m}\in R^{c\times 1\times 1}$. For a given spatial fine feature $X^{w}\in R^{\left(c\times h\times w\right)}$, the *XW* channel is divided into *αC* channels and (1 − *α*)*C* channels, where 0 ≤ *α* ≤ 1. Subsequently, a 1 × 1 standard convolution is employed to compress the feature map’s channels, further enhancing computational efficiency. Following this, by controlling the squeeze ratio *r* to adjust the size of CUR feature channels after performing segmentation and compression operations, the spatially refined feature *X*^*w*^ is partitioned into upper part *X*_*UP*_ and lower part *X*_*low*_. Notably, *X*_*UP*_ functions as a rich feature extractor inputted into the upper transformation stage; group-wise convolution (GWC) and point-wise convolution (PWC) are utilized to replace standard *K* × *K* convolutions for extracting features with reduced computational cost. Meanwhile, PWC is applied to *X*_*low*_ in order to generate a feature map with shallow hidden details.

$$\begin{array}{c} \beta_{1}=\frac{e^{s_{1}}}{e^{s_{1}}+e^{s_{2}}},\;\beta_{2}=\frac{e^{s_{2}}}{e^{s_{1}}+e^{s_{2}}} \end{array}$$ (4)

$$\begin{array}{c} \gamma =\beta_{1}\gamma_{1}+\beta_{2}\gamma_{2} \end{array}$$ (5)

Subsequently, *S*_1_ and *S*_2_ are concatenated, and channel-level soft attention operations are employed to generate feature importance vectors *β*_1_ and *β*_2_. Ultimately, guided by the feature importance vectors, *Y*_1_ and *Y*_2_ are fused to obtain the channel-refined features *β*_1_ and *β*_2_. The computational method is illustrated in Eqs 4 and 5.

**Fig 8:**
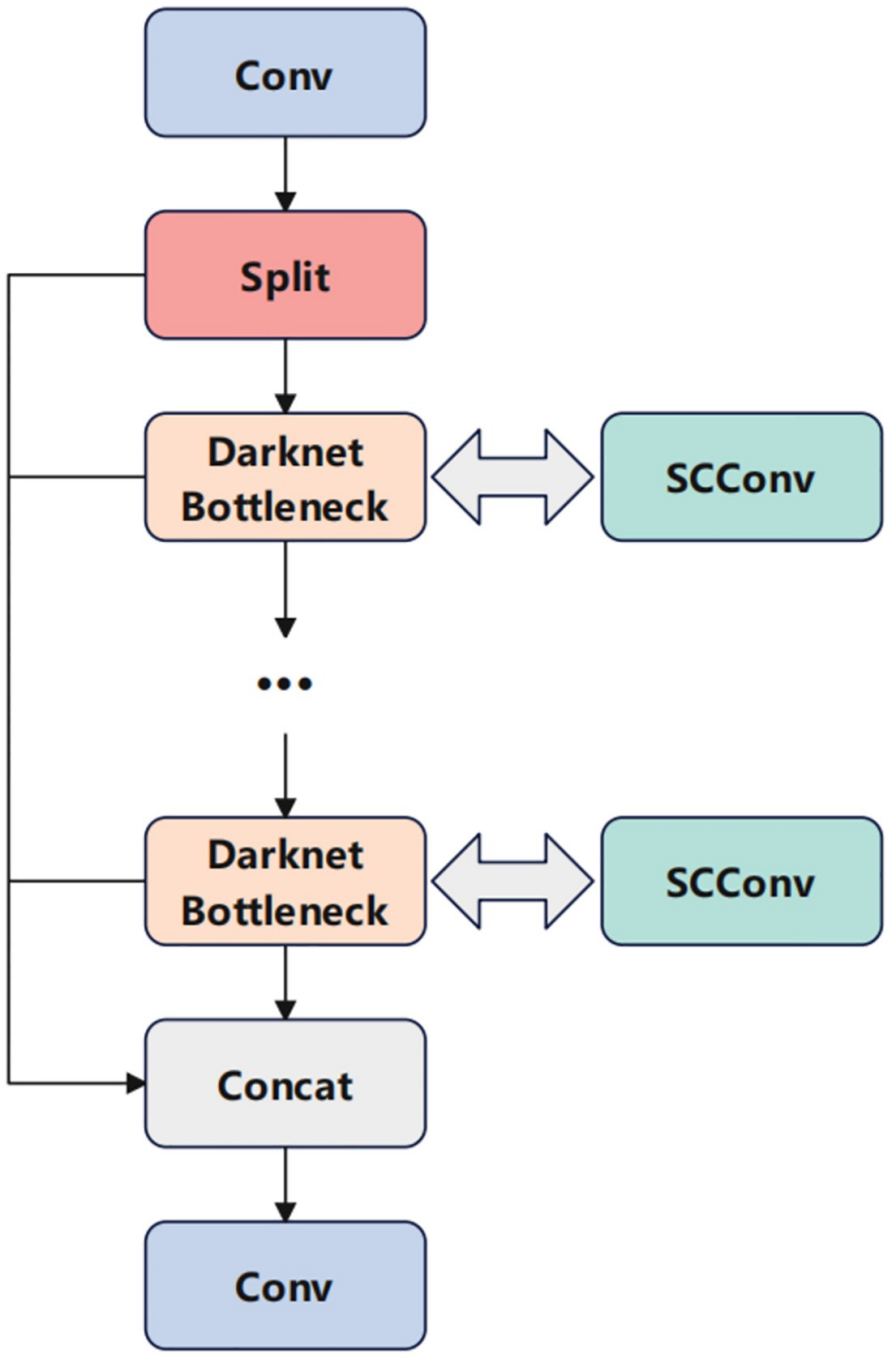
Structure of C2f_SCConv.

In this paper, a space and channel reconstruction convolutional module is employed to replace the original C2f network structure, as illustrated in Fig 8. This transformation consolidates all parameters into C2f_SCConv, resulting in significantly reduced memory usage while preserving high feature representation without compromising model accuracy.

#### SimAM

**Fig 9:**
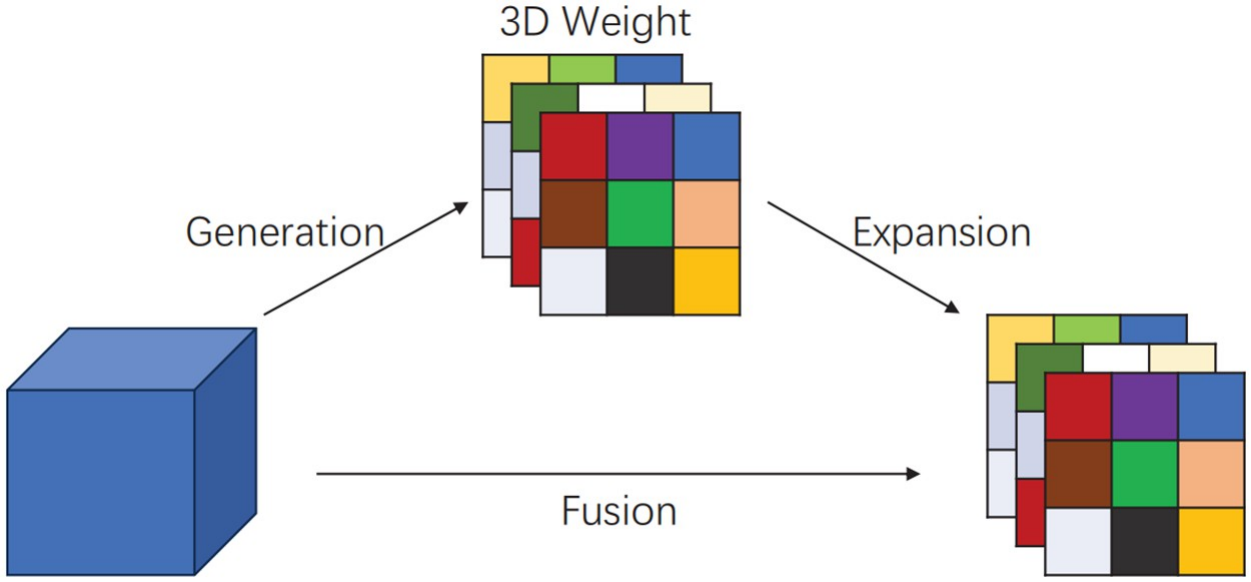
Structure of SimAM attention.

The SENet [25] and CBAM [26] models exhibit certain drawbacks when compared to SimAM. SENet requires a significant amount of computation and parameters, focusing primarily on channel relationships while neglecting spatial information. Similarly, the calculation complexity and parameter count of CBAM are higher, leading to longer training times. In contrast, SimAM [27] significantly enhances the model’s feature representation capability by efficiently addressing both spatial and channel information with its streamlined structure. AS shown in Fig 9, the SimAM attention does not require additional learning parameters. Instead, it deduces the three-dimensional attention weight through feature mapping of the feature map, effectively capturing target defect features while fully utilizing three-dimensional data information. It dynamically learns and utilizes similarity information between targets to accurately determine similarity indicators of various features. The minimum energy is calculated according to optimization of the energy function defined in Eq 6:

$$\begin{array}{c} e_{t}\left(w_{t},b_{t},y,x_{i}\right)=\frac{1}{M-1}\sum_{i=1}^{M-1}[{\left(-1-\left(w_{t}x_{i}+b_{t}\right)\right)}^{2}+{\left(1-\left(w_{t}t+b_{t}\right)\right)}^{2}]+\lambda w_{t}^{2} \end{array}$$ (6)

The target neuron *t* is represented by a single channel in the input eigenvalue $X\in R^{\left(C\times H\times W\right)}$, where *e*_*t*_ is the energy function of each neuron, *x*_*i*_ represents other neurons in the same channel, *M* = *H* × *W*, *H* denotes the height of the input picture, *W* denotes the width of the input picture, *w*_*t*_ and *b*_*t*_ are the weight variable and offset variable respectively, and λ is the hyperparameter. By taking the partial derivative of the above function’s variable and substituting it into the original function, we can minimize energy as follows.

$$\begin{array}{c} e_{t}^{*}=\frac{4({\hat{\sigma }}^{2}+\lambda )}{{\left(t-\hat{\mu }\right)}^{2}+2{\hat{\sigma }}^{2}+2\lambda } \end{array}$$ (7)

$$\begin{array}{c} \hat{\mu }=\frac{1}{M}\sum_{i=1}^{M}x_{i},\;{\hat{\sigma }}^{2}=\frac{1}{M}\sum_{i=1}^{M}{\left(x_{i}-\hat{\mu }\right)}^{2} \end{array}$$ (8)

The smaller the value of the above energy function $e_{t}^{*}$, the more linearly separable the target neuron is from other neurons, and the more important the neuron is compared to other neurons. Meanwhile, a Sigmoid function is employed to enhance features in order to prevent *E* energy values from becoming too large. Finally, the SimAM module is optimized as $\tilde{X}=\text{sigmoid}(\frac{1}{E})⊙X$, where *E* represents the sum of minimum energies across all spatial and channel dimensions.

In this equation, neuronal importance levels are obtained by $1/e_{t}^{*}$ and weighted accordingly. In this study, these levels are integrated into both LMD-YOLO’s backbone network and neck network to effectively refine characteristics within both channel and spatial domains. This integration significantly improves defect detection accuracy without increasing network complexity or computational resources.

#### SIoU

**Fig 10:**
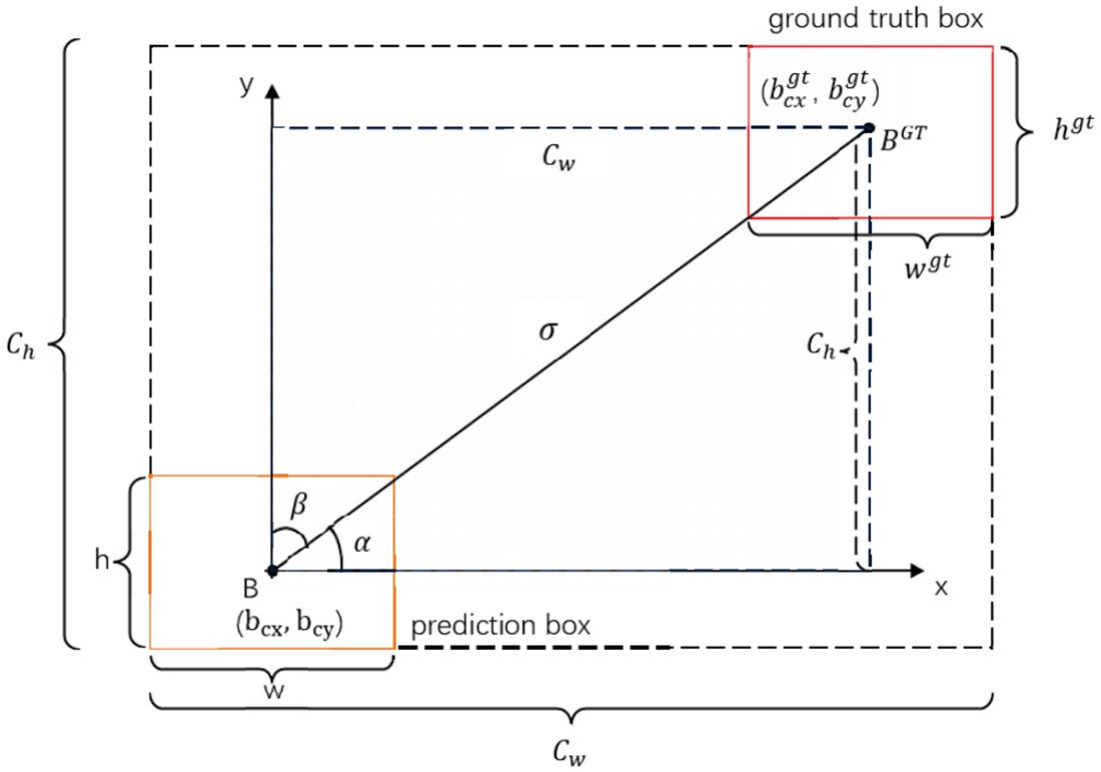
Siou loss function calculation diagram.

The benchmark model of YOLOv8 utilizes CIoU as the bounding box regression loss function, assessing the positional variance between the predicted and actual bounding box based on overlapping area, aspect ratio, and center point distance. However, it fails to consider the discrepancy between the predicted and target bounding box, leading to slow convergence and inadequate model accuracy during training. Therefore, this paper introduces SIoU [28] as a border regression loss function encompassing IoU loss, distance loss, shape loss, and angle loss. The scheme for calculation of angle cost contribution into the loss function, as shown in Fig 10. SIoU is defined in Eq 9:

$$\begin{array}{c} L_{\text{SloU}}=1-L_{loU}+\frac{\Delta +\Omega }{2} \end{array}$$ (9)

The formula for computing IoU loss is as follows: $IOU=\frac{|B\cap B^{gt}|}{|B\cup B^{gt}|}$ The prediction bounding box *B* and the real bounding box *B*^*gt*^ are denoted as such. The angle loss Λ is defined as the minimum angle between the line connecting the center point of the real bounding box and the target prediction bounding box, and the coordinate axis. Here, *σ* represents the distance between the centers of mass of the real bounding box and prediction bounding box, while *C*_*h*_ denotes the height difference between their centers of mass. The calculation formula is given by:

$$\begin{array}{c} \Lambda =\text{cos}(2\times (\text{arcsin}(\frac{c_{h}}{\sigma })-\frac{\pi }{4})) \end{array}$$ (10)

$$\begin{array}{c} \sigma =\sqrt{{\left(b_{c_{x}}^{gt}-b_{c_{x}}\right)}^{2}+{\left(b_{c_{y}}^{gt}-b_{c_{y}}\right)}^{2}} \end{array}$$ (11)

$$\begin{array}{c} c_{h}=max\left(b_{c_{y}}^{gt},b_{c_{y}}\right)-min\left(b_{c_{y}}^{gt},b_{c_{y}}\right) \end{array}$$ (12)

The distance loss Δ represents the distance between the center points of the prediction bounding box and the real bounding box, where the angle loss is directly proportional to the penalty cost. The calculation formula is as follows:

$$\begin{array}{c} \Delta =2-e^{-\gamma \rho_{s}}-e^{-\gamma \rho_{y}} \end{array}$$ (13)

$$\begin{array}{c} \rho_{x}={\left(\frac{b_{c_{x}}^{gt}-b_{c_{x}}}{C_{w}}\right)}^{2},\rho_{y}={\left(\frac{b_{c_{y}}^{gt}-b_{c_{y}}}{C_{h}}\right)}^{2},\gamma =2-\Lambda \end{array}$$ (14)

The coordinates $b_{c_{x}}^{gt}$ and $b_{c_{y}}^{gt}$ represent the true bounding box center, while $b_{c_{x}}$ and $b_{c_{y}}$ represent the predicted bounding box center. The formula for calculating the shape loss Ω is as follows:

$$\begin{array}{c} \Omega ={\left(1-e^{-W_{w}}\right)}^{\theta }+{\left(1-e^{-W_{h}}\right)}^{\theta } \end{array}$$ (15)

$$\begin{array}{c} W_{w}=\frac{|w-w^{gt}|}{max(w,w^{gt})} \end{array}$$ (16)

$$\begin{array}{c} W_{h}=\frac{|h-h^{gt}|}{max(h,h^{gt})} \end{array}$$ (17)

The variables *w* and *h* denote the width and height of the predicted bounding box, while *w*^*gt*^ and *h*^*gt*^ represent the width and height of the real bounding box.

#### Dysample

The Dysample module employs a unique dynamic upsampling method to address the challenges of detecting small insulator defects in low-quality images. Low-quality images often suffer from insufficient resolution, blurriness, and loss of detail, making it difficult to detect small defects. Traditional upsampling methods, such as nearest-neighbor interpolation and bilinear interpolation, use fixed rules for interpolation and cannot dynamically adjust based on the image content, making them ineffective at accurately recovering small defects in low-quality images. In contrast, Dysample leverages a content-aware sampling point generation mechanism, which adapts the sampling positions according to the content of the input feature map. It first applies bilinear interpolation to the input feature map, transforming it into a continuous spatial representation, and then dynamically generates offsets to adjust the sampling positions. This dynamic sampling process better captures image details, particularly for small defects that are easily overlooked in low-resolution images, significantly enhancing detection capability. Additionally, traditional kernel-based upsampling processes involve significant computational and parameter overhead, which is not conducive to developing lightweight network architectures.

**Fig 11:**
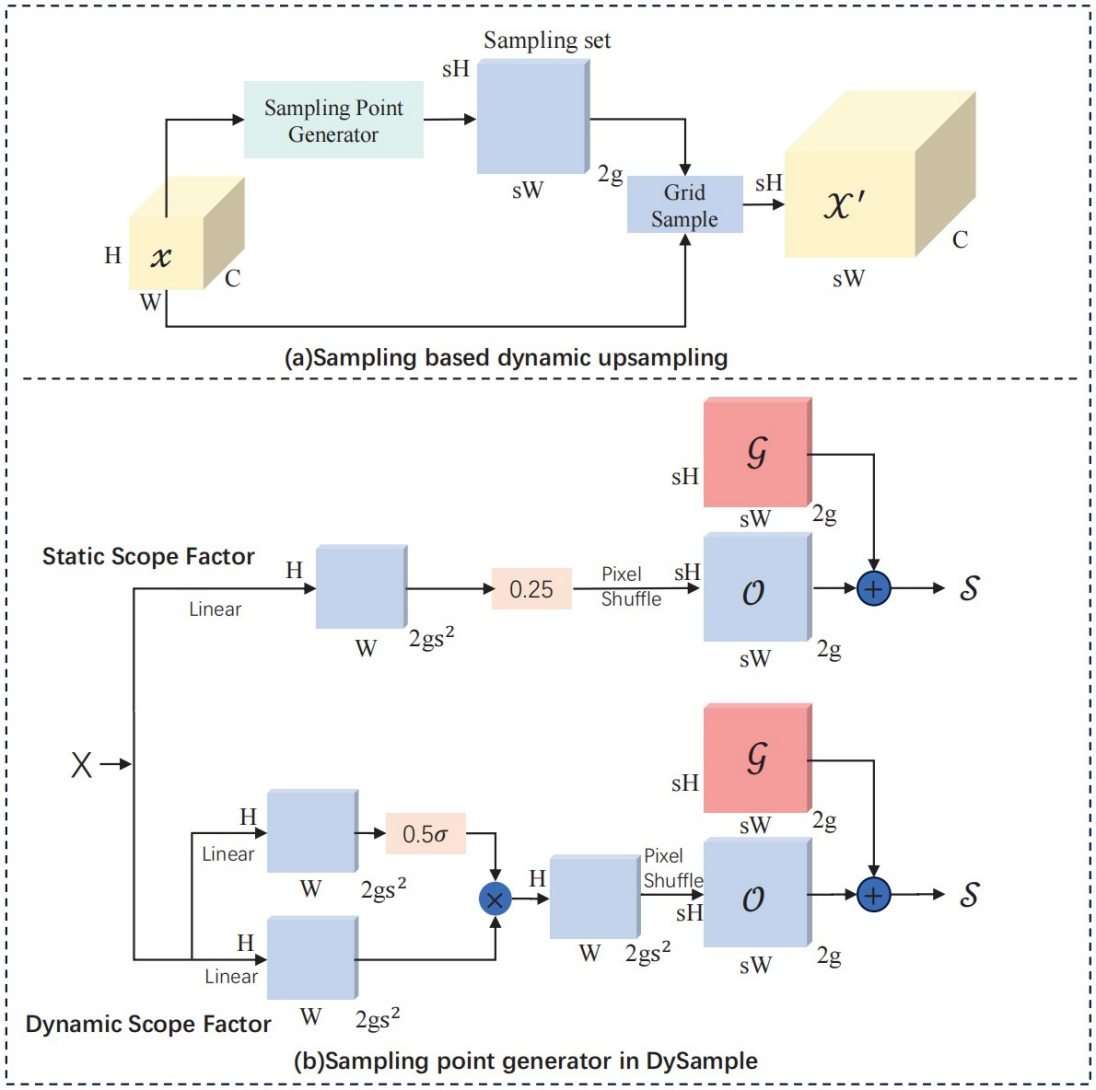
The input feature map *X*, the upsampled feature map *X*′, the generated offsets *O*, and the original grid *G* are depicted. In Fig 11(a), the sampling set is created by the sampling point generator, and the input feature map *X* is resampled according to this set using a grid sampling function. In Fig 11(b), the sampling set is the sum of the generated offsets *O* and the original grid positions *G*. The top shows the version with a “static Scope factor,” where offsets are produced through a linear layer. The bottom displays the version with a “dynamic Scope factor,” where a scope factor is generated first and then used to modulate the offsets. The symbol “*σ*” represents the sigmoid function.

In real grid inspection scenarios, small insulator defect pixels may cause image pixel distortion and other issues, leading to fine-grained detail loss and difficulties in feature learning for recognition tasks. In order to tackle this issue, this paper introduces Dysample [29]—a highly lightweight and efficient dynamic up-sampler specifically designed for enhancing defect detection in low-resolution images with smaller insulators. In terms of detection granularity, The Dysample significantly enhances the ability to capture fine image details by dynamically adjusting the position and range of sampling points, demonstrating particular effectiveness in detecting small defects with complex boundaries. Compared to traditional upsampling methods, Dysample mitigates inaccuracies caused by overlapping or irregularly distributed sampling points. By constraining the offset range, Dysample ensures a more uniform distribution of sampling points, thereby improving edge detection accuracy. This is especially crucial for precisely localizing small defects, as it enables more accurate representation of defect shapes and sizes. Moreover, Dysample introduces a grouping mechanism that dynamically adjusts the offset within each group, allowing for more flexible and precise movement of sampling points. This further strengthens the robustness and computational efficiency of the upsampling process. The mechanism also enables shared sampling point generation across different groups, optimizing computational resources. As a result, Dysample exhibits superior performance in various dense prediction tasks, particularly in improving detection accuracy and edge resolution for small defects, outperforming traditional methods. Fig 11 illustrates the process of generating the sampling set and the design of the dynamic upsampling module in DySample. The dynamic up-sampler adjusts the up-sampling parameters of the input feature map using point sampling method. By learning the dynamic structure of the input sequence and generating new data points during sampling on the feature graph without time-consuming dynamic convolution operation or additional network parameters, Dysample enhances image resolution without introducing a large computational load, resulting in improved model efficiency and performance at a reduced computational expense owing to its decreased resource demands.

## Results and discussion

### Experiment settings and parameter settings

The parameters of the experiments performed in this paper are shown in Table 2.

**Table 2:** Experimental hyperparameter setting.

Hyperparameters	Values
Epochs of Train	400
Warmup epochs	10
Batch Size	16
Learning rate	0.001
Momentum	0.937
Optimizer	Adam
Images Size	640 × 640

The experimental hardware and software environments utilized in this paper are shown in Table 3.

**Table 3:** Experimental software and hardware configuration.

Item	Specification
Deep Learning Framework	PyTorch 1.13.1
Data Processing Environment	Python 3.8.19
Operating System	Windows 11
GPU Parallel Computing Library	CUDA 11.1
GPU	NVIDIA RTX 3070 Laptop GPU
CPU	AMD Ryzen 7 5800H

### Evaluation metrics

In order to quantitatively confirm the efficacy of the proposed network, we have incorporated four assessment criteria: average precision (AP), mean average precision (mAP), Giga Floating-point Operations Per Second (GFLOPs), and parameters. The AP is calculated using Precision (P) and Recall (R). MAP was selected as the performance evaluation metric for the models. The formulas are as follows:

$$\begin{array}{c} P=\frac{T_{P}}{T_{P}+F_{P}} \end{array}$$ (18)

$$\begin{array}{c} R=\frac{T_{P}}{T_{P}+F_{N}} \end{array}$$ (19)

Where TP represents the number of samples accurately classifying positive instances as insulator-labeled; Fn denotes the count of samples erroneously identifying positive instances as negative, i.e., misclassifying non-insulator-labeled samples as insulator-labeled; and FP signifies the count of samples mistakenly identifying negative instances as positive, i.e., incorrectly categorizing insulator-labeled samples. Accuracy measures the evaluation algorithm’s ability to identify positive instances from the dataset, while recall assesses its capability to recognize positive instances from the dataset. mAP denotes the average precision across all target categories. A higher mAP value indicates better overall performance of the current algorithm. The calculation for mAP is as follows:

$$\begin{array}{c} AP=\int_{0}^{1}p\left(r\right)dr \end{array}$$ (20)

$$\begin{array}{c} mAP=\frac{1}{n}\sum_{i=1}^{n}AP_{i} \end{array}$$ (21)

The GFlops metric serves as a crucial indicator for assessing the computational performance of the YOLO model. It quantifies the number of billion floating-point operations that a model can execute per second, with higher values indicating greater computational intensity. This measure facilitates comparisons of efficiency and computational expenses across different models. Parameters denote the count of trainable variables within the model, encompassing both weights and biases. The number of parameters reflects the complexity and storage requirements of the model. A higher number of parameters enhances the expressiveness of the model. The more parameters, the better the expressiveness of the model. In YOLO models, the number of parameters is often used to assess model size and resource requirements.

### Ablation experiments

To assess the performance enhancement brought by each modified module in the LMD-YOLO model compared to the original YOLOv8 network, four enhancement methods (C2f_SCConv, SimAM attention mechanism, SIoU loss function, and Dysample) were successively integrated into the original network model. The subsequent experiments in this study are mainly based on the YOLOv8n as the base model for enhancement and innovation. The ablation experiment utilized identical datasets and training strategies, with experimental results detailed in Table 4. In Experiment 2, C2f modules within both the backbone and neck networks of YOLOv8 were substituted with C2f_SCConv to improve detection accuracy while reducing network parameters. Expanding on Experiment 2, Experiment 3 introduced SimAM attention modules into both the backbone and neck networks of YOLOv8 to enhance feature extraction capabilities without introducing additional parameters. This also improved the model’s ability to capture local information on insulator defects. Experiment 4 replaced CIoU with SIoU based on findings from Experiment 3. Finally, in Experiment 5, a lightweight and efficient dynamic up-sampler was added onto the foundation laid by Experiment 4 to strengthen detection capabilities for low-resolution images and small insulator defects. Experiment 6 exclusively employs SimAM, achieving a mean Average Precision of 86.8%. This underscores the significance of SimAM attention in enhancing feature extraction and improving detection accuracy. Experiment 7 concentrates solely on the SIoU loss function, resulting in an mAP@0.5 of 86.1%. This indicates that while SIoU contributes to better localization of bounding boxes, its effect on overall detection precision may be somewhat limited without the integration of additional complementary modules. In Experiment 8, only the Dysample module is utilized, yielding an mAP@0.5 of 86.0%. This outcome confirms that dynamic upsampling strategy provides certain benefits in feature alignment, potentially improving the model’s ability to capture fine details.however, combining it with other modules could potentially enhance performance further.

**Table 4:** Ablation experiments.

Experiment Number	C2f_SCConv	SimAM	SIoU	Dysample	Params	GFLOPs	mAP@0.5(%)
1	-	-	-	-	3006428	8.1	85.9
2	✓	-	-	-	2254524	6.2	86.7
3	✓	✓	-	-	2254524	6.2	87.1
4	✓	✓	✓	-	2254524	6.2	87.4
5	✓	✓	✓	✓	2266876	6.2	87.9
6	-	✓	-	-	3006428	8.1	86.8
7	-	-	✓	-	3006428	8.1	86.1
8	-	-	-	✓	3018780	8.1	86.0

The model introduced in this paper effectively decreases the quantity of parameters and floating-point calculations by substituting C2f_SCConv, offering a new approach for deploying models on embedded devices. Furthermore, with the introduction of attention mechanism, replacement loss function, and dynamic sampling operator, the detection accuracy and performance on insulators are further enhanced. With a 25% reduction in size compared to the original model, the LMD-YOLO model achieves a 2% increase in average accuracy. To further analyze the performance gains of the model on various defect types, we aligned the experiments in Table 5 with the corresponding experiment numbers in Table 4. This correspondence allows for a more detailed comparison, helping to identify the specific contributions of individual components to defect detection improvements. LMD-YOLO model attains the highest MAP of 87.9%. Among the various types of insulator defects, the broken state demonstrates the most significant improvement in our model. Notably, in Experiment 5, the AP for intact defects reached 88.2%, reflecting an enhancement of 8.3% over the baseline model, this marks the highest improvement across all defect categories. This advancement can be primarily attributed to the integration of attention mechanisms and dynamic sampling operators within the model, which enable it to focus more accurately on critical regions and enhance its capacity to learn from complex samples. Furthermore, the model exhibited commendable performance on normal insulator types and shedding targets, achieving APs of 96.7% and 85.1%, respectively. Although there was a slight decline in performance under occlusion conditions, resulting in absent insulator AP of approximately 88.5%, representing a decrease of 1.8% from the benchmark model, it still demonstrated robust detection capabilities. Compared to Experiment 1, Experiment 6 demonstrates a 0.9% increase in mAP@0.5, primarily attributed to enhancements in the detection of Broken (75.4%) and Shedding (85.9%) defects. This indicates that SimAM effectively improves feature extraction for these challenging defect types. Experiment 7 reveals a modest increase of 0.2% in mAP@0.5, suggesting that the SIoU loss function provides slight improvements in bounding box localization; however, its overall impact remains limited when applied independently. In Experiment 8, there is a marginal increase of 0.1% in mAP@0.5, indicating that the dynamic upsampling strategy yields minor improvements by better aligning features; nonetheless, it may not be sufficient on its own to significantly enhance performance.

**Table 5:** Ablation experiments.

Experiment Number	AP(%)				mAP@0.5(%)
	Insulator	Absent	Broken	Shedding	
1	96	90.3	72.9	84.6	85.9
2	96	88.2	75.9	86.8	86.7
3	96.7	89.3	77.3	85.3	87.1
4	96	92	76.7	85.1	87.4
5	96.7	88.5	81.2	85.1	87.9
6	96.0	90.1	75.4	85.9	86.8
7	95.8	87.2	75.6	85.9	86.1
8	95.4	87.5	77.4	83.9	86.0

In summary, the LMD-YOLO model has shown marked improvements across various insulator types and defect states, with particularly notable enhancements observed in defect states. These advancements are largely due to the incorporation of attention mechanisms, alternative loss functions, and dynamic sampling operators that collectively contribute to improved detection accuracy and overall performance of the model. Improved model demonstrates a significant enhancement in insulator multi-defect detection performance, accompanied by reduced model size and computing resources. It fulfills the requirements for lightweight deployment with improved accuracy.

**Fig 12:**
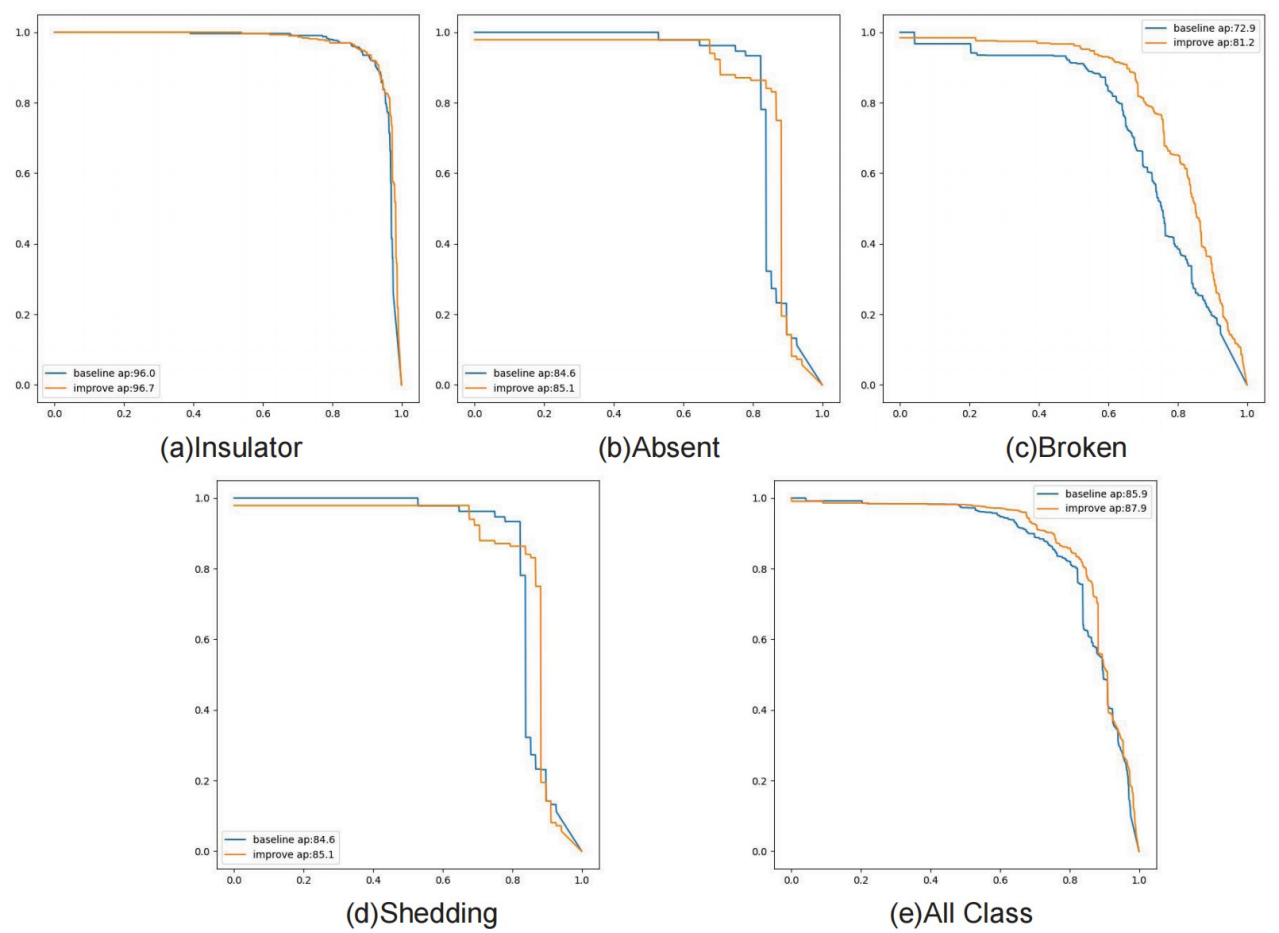
The PR curve charts for each class before and after model improvement.

By Analyzing the PR curve, it is commonly observed that as the recall rate increases, the accuracy rate decreases, and vice versa. A PR curve closer to the upper right corner indicates that the model can ensure high accuracy and recall rates when predicting insulator defects, leading to more precise predictions. In the PR curve, ‘Insulator’, ‘Absent Insulator’, ‘Broken Insulator’, ‘Shedding Insulator’ and ‘All Class’ of YOLO v8 are 96%, 90.3%, 72.9%, 84.6% and 85.9% respectively; LMD-YOLO models were 96.7%, 88.5%, 81.2%, 85.1% and 87.9% respectively, improving by 0.7%, -1.8%, 8.3%, 0.5% and 2%, as shown in Fig 12. Although the ap value of Absent Insulator decreased by 1.8%, the ap value of the total class increased. It can be concluded that the enhanced network LMD-YOLO has improved effectiveness in detecting multiple insulator defects by increasing mAP by 2%.

**Fig 13:**
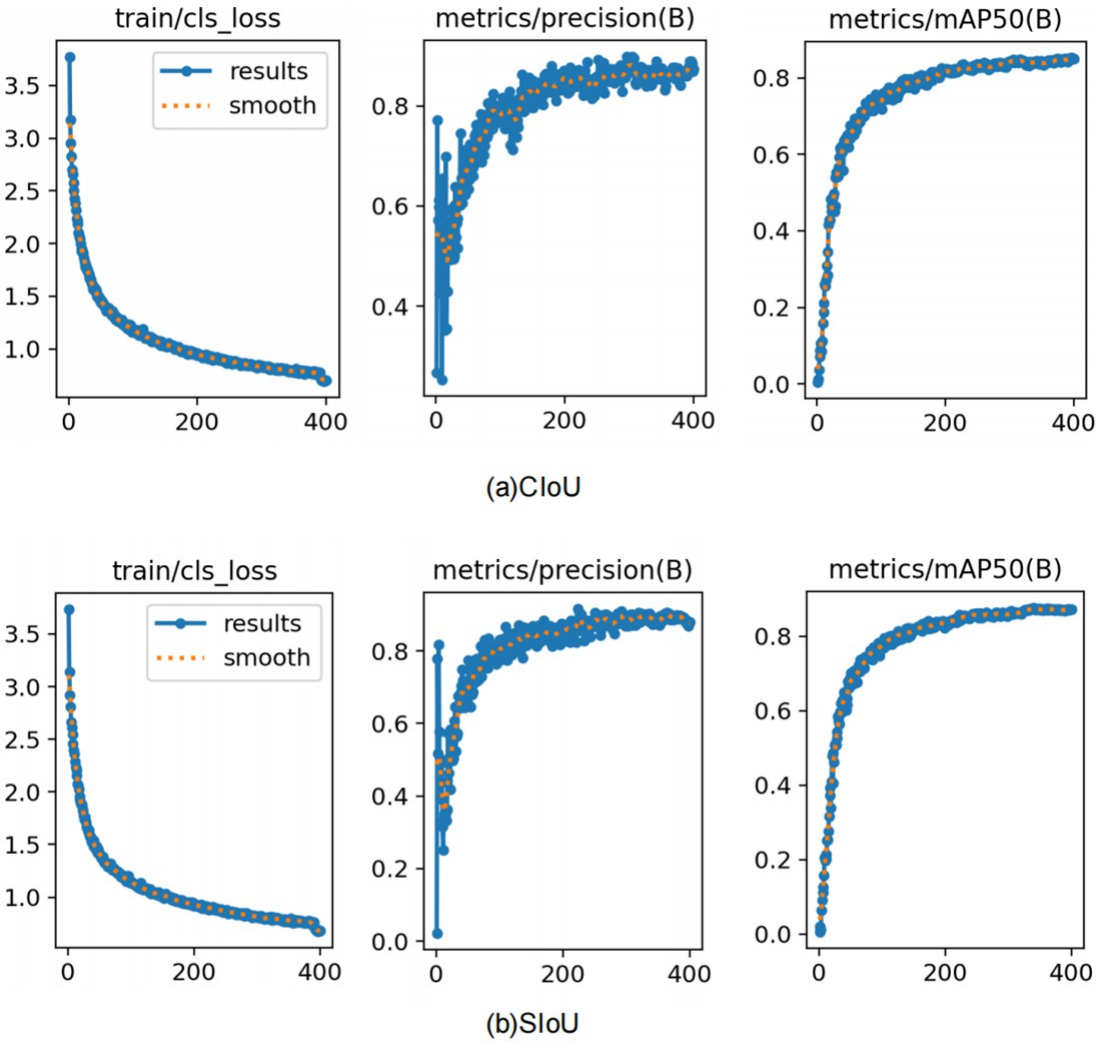
Comparison of SIoU and CIoU loss functions: Classification loss, precision, and mAP50 convergence performance.

As illustrated in the Fig 13, the SIoU loss function demonstrates superior performance compared to the CIoU loss function regarding both convergence speed and defect detection accuracy. While both functions exhibit a decreasing trend in classification loss throughout training, SIoU presents a smoother and more stable loss curve due to its incorporation of angle differences between predicted and ground-truth bounding boxes. The geometric characteristics inherent to SIoU facilitate more effective alignment of bounding boxes, thereby providing clearer gradient signals during the early stages of training and promoting faster convergence. Furthermore, although both CIoU and SIoU achieve commendable accuracy levels, SIoU displays a more stable accuracy curve, indicating an enhancement in object localization consistency. Notably, SIoU achieves higher mAP50 scores across multiple training cycles, an essential factor for detecting small and complex-shaped defects such as broken insulators.

Considering all these aspects, it is evident that SIoU offers a more comprehensive metric for loss calculation by integrating angle adjustments along with other geometric factors. This enables the model to predict bounding boxes with greater precision in low-quality images while significantly enhancing defect detection performance.

The implementation of the SimAM attention mechanism results in a Recall value that peaks at 92.9%, significantly surpassing that of other attention mechanisms, as shown in Table 6. By assigning weights to each pixel, SimAM effectively emphasizes spatial regions pertinent to the target while suppressing irrelevant background information. This capability enables the network model to more accurately identify small defects, even those partially obscured by complex backgrounds, thereby markedly enhancing defect detection rates. Consequently, complex backgrounds are less likely to lead to missed detections, SimAM adeptly extracts spatial features associated with defects from intricate scenes. However, SimAM exhibits a lower Precision value of 85.1%. This indicates that although this mechanism successfully identifies a greater number of defects, it may also result in an increased incidence of false positives. Despite these potential false positives, the overall architecture of LMD-YOLO facilitates an effective balance between Precision and Recall. For instance, this balance can be achieved through non-maximum suppression during post-processing to filter out low-confidence false positive boxes or by employing the Siou loss function. Such strategies enable SimAM to enhance Recall without substantially compromising Precision. ELA [30] improves recall (88.5%) but has limited impact on mAP@0.5. The SE attentional mechanism performed best in Precision(89.7%), indicating that it detected defects more accurately, but was weak in Recall (79.6%), indicating some problems in missed detection. In addition, the EMA [31] attention mechanism, while showing the same performance as the SE on Recall, had a slightly lower Precision of 89.2%.

**Table 6:** Ablation experiments.

Yolov8n	ELA	EMA	SE	SimAM	Precision	Recall	mAP@0.5(%)
✓	-	-	-	-	88.5	80.5	85.9
✓	✓	-	-	-	88.9	88.5	86.0
✓	-	✓	-	-	89.2	79.6	86.6
✓	-	-	✓	-	89.7	79.6	86.5
✓	-	-	-	✓	85.1	92.9	86.8

In conclusion, MAP@50, as a comprehensive evaluation index, can measure the overall performance of each model in multiple dimensions. SimAM performs best on MAP@50 at 86.8%, ELA improves mAP@0.5 by only 0.1%, indicating its limited contribution to overall detection performance despite enhancing recall. EMA and SE mechanisms at 86.5% and 86.6%, respectively, indicating that SimAM achieves a good balance between accuracy and recall.

### Comparison of detection performance

To validate the effectiveness of the LMD-YOLO improvement, we selected comparative experiments on a custom dataset using the YOLO series as one-stage lightweight target detection network, and the two-stage target detection networks Faster-RCNN and Cascade-RCNN, as illustrated in the Table 7. The approach proposed in this paper outperforms other lightweight models in terms of parameter count and surpasses the two-stage network in detection accuracy. Two-stage networks such as Faster-RCNN and Cascade-RCNN lag behind LMD-YOLO by approximately 2.4% and 3.2%, respectively, in terms of detection accuracy, while their parameter counts are about 18 times and 30 times higher than that of LMD-YOLO. When compared with other lightweight models, LMD-YOLO exhibits distinct advantages across various evaluation metrics. While reducing parameter count, LMD-YOLO ensures improved detection accuracy, indicating its superior precision and ease of deployment in UAV target detection scenarios.

**Table 7:** Contrasting experiment results.

Model	Params(M)	GFLOPs	mAP@0.5(%)
Faster-RCNN	41.39M	208	85.5
Cascade-RCNN	69.29M	236	84.7
Yolov5n	2.5M	7.1	85.7
Yolov3n-Tiny	12.1M	18.9	84.9
Yolov7n-Tiny	6.0M	13.2	85.6
Ours	**2.27M**	**6.2**	**87.9**

### Visualization

**Fig 14:**
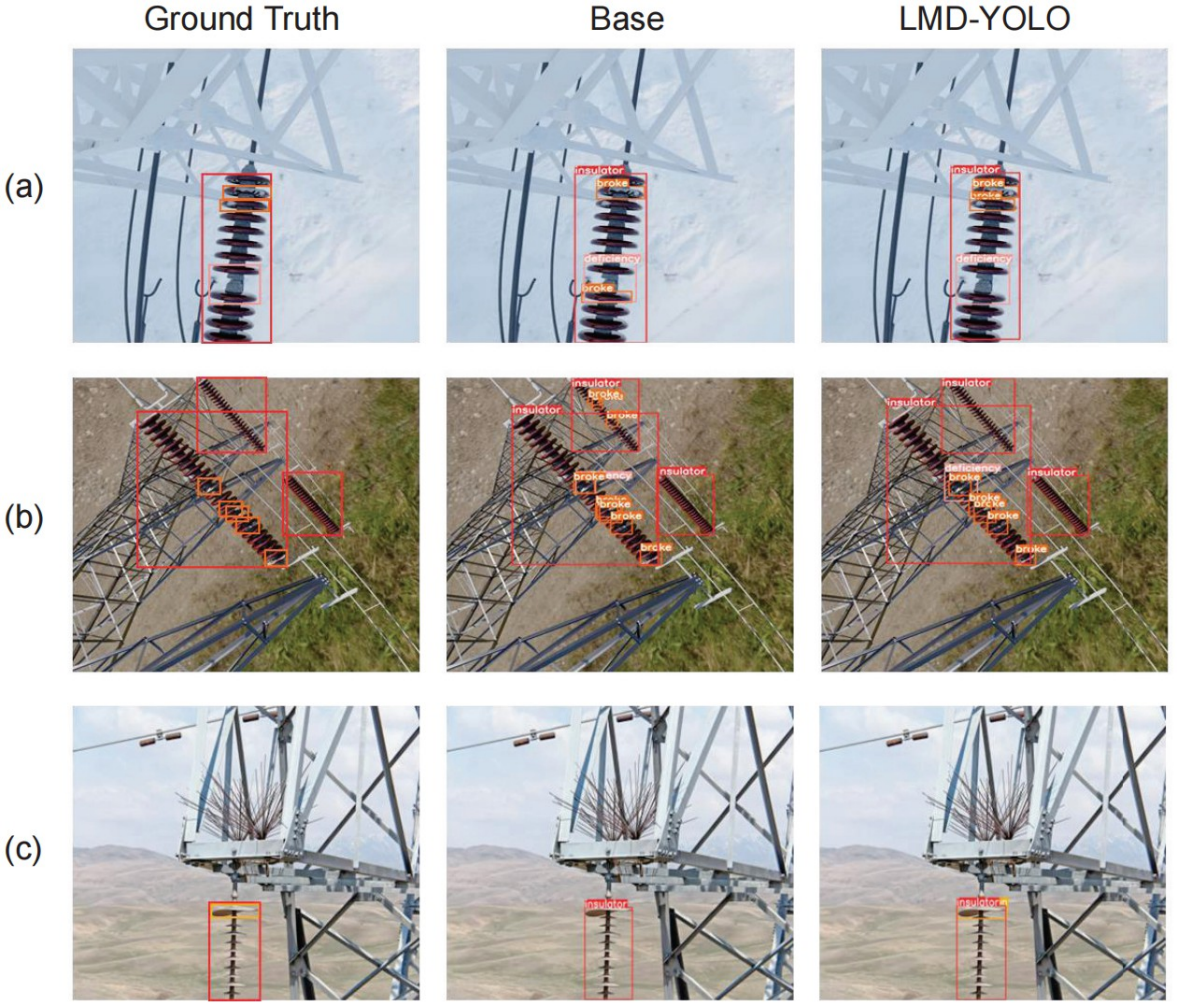
Visual assessments across three real scenarios: Scenes with backgrounds similar to defects (a), scenes with small targets (b), and simple scenes (c). Ground Truth represents labels for actual defects; Base represents detection results using YOLOv8.

In this part, we offer a visual evaluation of LMD-YOLO in actual transmission line situations and various environmental circumstances (Fig 14). More specifically, we classify three different types of real-life settings: scenes with backgrounds similar to defects, scenes with small targets, and simple scenes. In Fig 14(a), due to the insulator damage closely resembling the background color, YOLOv8 exhibits a missed detection and false detection, highlighting LMD-YOLO’s superior generalization in identifying insulator images under challenging shooting conditions. Additionally, in Fig 14(b), YOLOv8 demonstrates subpar performance in detecting small objects by erroneously identifying damaged positions of small insulators; conversely, LMD-YOLO excels in its detection capabilities and displays heightened sensitivity towards small insulator breakage objects, indicating the efficacy of the SimAM module. Finally, in Fig 14(c), LMD-YOLO successfully identifies the location of an insulator loss defect while YOLOv8 struggles to do so and experiences numerous instances of missed detections.

## Conclusions

To address the challenges of complex backgrounds and deployment constraints on resource-limited devices, we enhanced the YOLOv8 model by introducing multiple improvements, culminating in the development of the lightweight defect detection model, LMD-YOLO. By incorporating the Scconv module, the network’s sensitivity to spatial details and channel features is enhanced. Additionally, with the integration of the SimAM attention mechanism and the optimization of the CIoU loss function to the SIoU loss function, detection accuracy is further improved. The improved network significantly reduces both computational cost and parameter count, resulting in enhanced performance. Detection accuracy increases by 2%, while the parameter count decreases by 25%. Experimental results demonstrate that LMD-YOLO can effectively identify various types of defects in complex power distribution network environments and meet diverse insulator defect detection requirements. While improving detection accuracy, it also reduces demands on computational and storage resources, making deployment feasible on resource-constrained embedded devices.

Future research will be directed towards several areas, including optimizing the runtime for faster detection speed while ensuring accuracy, which is crucial for UAV detection tasks. Additionally, future work will consider integrating insights from Li, Y et al. [32] proposed a federated learning approach for detecting false data injection attacks in smart grids, This will help ensure that the LMD-YOLO model continues to perform reliably while also safeguarding against potential security threats. Furthermore, we will strive to improve the network’s robustness in challenging environmental situations, including fog, rain, and low-light conditions, to guarantee consistent performance in practical detection scenarios.
